# Insights Into Cystic Fibrosis Gene Mutation Frequency, Clinical Findings, and Complications Among Pakistani Patients

**DOI:** 10.7759/cureus.48564

**Published:** 2023-11-09

**Authors:** Asaf Syed, Anurag Rawat, Umer Bin Tariq, Ihteshamul Haq, Beenish Naz, Abrar Hussain, Mehdi Maqsood, Arsalan Rasheed

**Affiliations:** 1 Medicine and Surgery, Ayub Medical College, Abbottabad, PAK; 2 Interventional Cardiology, Himalayan Institute of Medical Sciences, Dehradun, IND; 3 Department of Medicine, Nawaz Sharif Medical College, University of Gujrat, Gujrat, PAK; 4 Department of Biotechnology and Genetic Engineering, Hazara University, Mansehra, PAK; 5 Pharmacology and Therapeutics, Khyber Medical University, Peshawar, PAK; 6 Biological Sciences, International Islamic University, Islamabad, PAK; 7 Internal Medicine, Khyber Medical College, Peshawar, PAK; 8 Molecular Biology and Genetics, Abdul Wali Khan University Mardan, Mardan, PAK

**Keywords:** pakistan, complications, clinical findings, gene mutations, cftr, cystic fibrosis

## Abstract

Background

Cystic fibrosis (CF) is a genetic disorder with diverse symptoms. Understanding its genetic basis and prevalence is crucial for effective management and treatment.

Objective

The study aimed to provide comprehensive insights into the frequency of CF gene mutations, clinical presentations, and complications among the Pakistani population.

Methodology

A cohort comprising 892 patients, ranging in age from 18 to more than 40 years, was selected on the basis of clinical and genetic criteria for the diagnosis of CF. Polymerase chain reaction (PCR) was used to look for 34 variants in the CFTR gene in blood samples. Statistical analysis, which included figuring out the number of mutations, the average age of diagnosis, and the genetic diversity of the samples, was performed to analyze the percentage of patients with specific mutations, offering insights into the genetic diversity.

Results

In our comprehensive analysis of 892 patient samples, 77.47% (n=691) displayed consanguinity, indicating a family history. The prevailing symptoms included chronic cough (88.67%; n=791), recurrent respiratory infections (76.68%; n=684), and fatigue (73.76%; n=658). The major complications comprised pulmonary infections (22%; n=197), cystic fibrosis-related diabetes (21%; n=187), and malabsorption (20%: n=178). A paired t-test revealed a mean difference of 5.750 with a standard deviation of 9.147, a 95% confidence interval from -0.061 to 11.561, a t-value of 2.178 with 11 degrees of freedom, and a two-tailed p-value of 0.052, suggesting a potential trend towards significance. Nevertheless, the asymptotic significance values of 1.000 and 0.998 for both groups indicate no significant difference. Furthermore, the study identified 12 cystic fibrosis gene mutations, with F508del and N1303K being the most prevalent.

Conclusion

This research revealed significant consanguinity, confirmed typical CF symptoms, and identified common complications and prevalent CFTR gene mutations (with F508del and N1303K being the most common), providing insights for genetic guidance and treatment in the Pakistani community.

## Introduction

Cystic fibrosis (CF) is a hereditary disorder characterized by the malfunctioning of the cystic fibrosis transmembrane conductance regulator (CFTR) gene [1,2]. CFTR gene mutations lead to the production of defective CFTR proteins, resulting in the accumulation of thick mucus in various organs, primarily affecting the lungs, pancreas, and digestive system [3-5]. CF is a global health issue affecting individuals of all ethnicities and geographical locations [6]. Approximately 70,000 to 100,000 people are thought to be affected by CF globally [7]. Limited data exist regarding the exact prevalence of CF in the Pakistani population, and it remains poorly documented [8]. The frequency of CF gene mutations, along with the clinical manifestations and complications, varies across populations [9]. Therefore, understanding the specific genetic profile, clinical findings, and associated complications within the population of Pakistan is crucial for effective management and improved outcomes. The study aimed to unravel insights into CF gene mutation frequency, clinical findings, and complications within the population of Pakistan.

Significance

The findings of this study will not only contribute to the existing knowledge regarding CF in this specific population but also provide valuable insights for healthcare professionals, enabling them to develop targeted approaches for diagnosis, management, and genetic counseling, ultimately improving the quality of care and outcomes for CF patients in Pakistan.

## Materials and methods

Study design

The retrospective study was conducted in the Department of Pulmonology at the Pakistan Institute of Medical Sciences (PIMS), Islamabad, over a period of two years, from January to December 2022.

Ethical statement

The study was approved by the research ethics committee of the International Islamic University, Islamabad, Pakistan. Before participating in the trial, each patient provided a signed consent form.

Inclusion and exclusion criteria

The inclusion criteria for this study entail individuals aged 18 to 40 who have received a confirmed diagnosis of CF. Participants who were eligible had to have full medical records that included a detailed medical history, hospitalization records, a list of current symptoms, a history of medications, treatment plans, relevant pulmonary function test results, as well as clinical and genetic information. Exclusion criteria involve patients with incomplete records or those who declined participation or lacked informed consent.

Sample size

In this retrospective investigation, 892 blood samples from individuals who had CF were examined. The age of the patients in this study ranged from below 18 to above 40 years.

Sampling technique

Blood samples collected from patients were sent to the Islamabad Diagnostic Centre (IDC) in Pakistan. To isolate DNA from these samples, the chloroform method was employed. Subsequently, polymerase chain reaction (PCR) was carried out to assess 34 mutations in the CFTR gene. Molecular analysis was conducted using the CF stripAssay® kit provided by ViennaLab Diagnostics, GmbH, based in Austria. During PCR, DNA was amplified using biotinylated primers, resulting in the generation of amplified DNA fragments. The thermal cycling process commenced with an initial step at 95°C for 3 minutes, followed by 40 cycles of denaturation at 95°C for 3 seconds and annealing/extension at 60°C for 30 seconds. These fragments were then subjected to a hybridization process in which they interacted with strips containing an array of parallel lines, each with allele-specific probes designed to detect and bind specific DNA sequences associated with distinct CF mutations. To visualize and identify the bound sequences on the strips, streptavidin-alkaline-phosphatase and a color substrate were utilized. This combination allowed for the detection of specific mutations within the patient's DNA. For comprehensive mutation analysis, two strips were employed for each patient's sample. Strip A covered 18 mutations while strip B encompassed 16 mutations. To ensure the accuracy and reliability of the assay, a positive reaction control line was included at the top of each test strip, confirming the proper functioning of the conjugate solution and color developer.

Statistical analysis

Statistical analysis was performed in SPSS (version 27.0; IBM Corp. Armonk, NY). Descriptive tests were used to calculate the mean patient age and assess genetic variability. Chi-square tests were conducted to investigate the association between certain genetic variants and clinical manifestations while paired samples T-tests were utilized to compare mean differences between heterozygous gene mutant and homozygous gene mutant groups, providing a comprehensive understanding of the genetic and clinical aspects of CF in the study. P-value <0.05 was considered significant.

## Results

In this retrospective investigation, a total of 892 patient samples were analyzed during the course of this study. The study analyzed 892 patients with CF in the Pakistani population, revealing a gender distribution of 523 males and 369 females. While no sex bias was found in the genetic distribution of CF, potential sex-based differences were identified in certain clinical features. Regarding age group, the 18-40 years category constitutes the largest proportion, with approximately 43.83% of the patients (n=391) falling within this age range. Consanguinity was prevalent in the majority of cases (n=691; 77.47%), indicating a blood relation between parents (Table 1).

**Table 1:** Demographic and genetic characteristics of cystic fibrosis patients in Pakistan

Variables	Patients Number (n)	Percentage (%)
Gender		
Male	523	58.63
Female	369	41.37
Age Group		
Below 18 years	187	20.96
18-40 years	391	43.83
Above 40 years	314	35.21
Consanguinity		
Positive	691	77.47
Negative	201	22.53
Family history		
Positive	168	18.84
Negative	724	81.16

**Figure 1:**
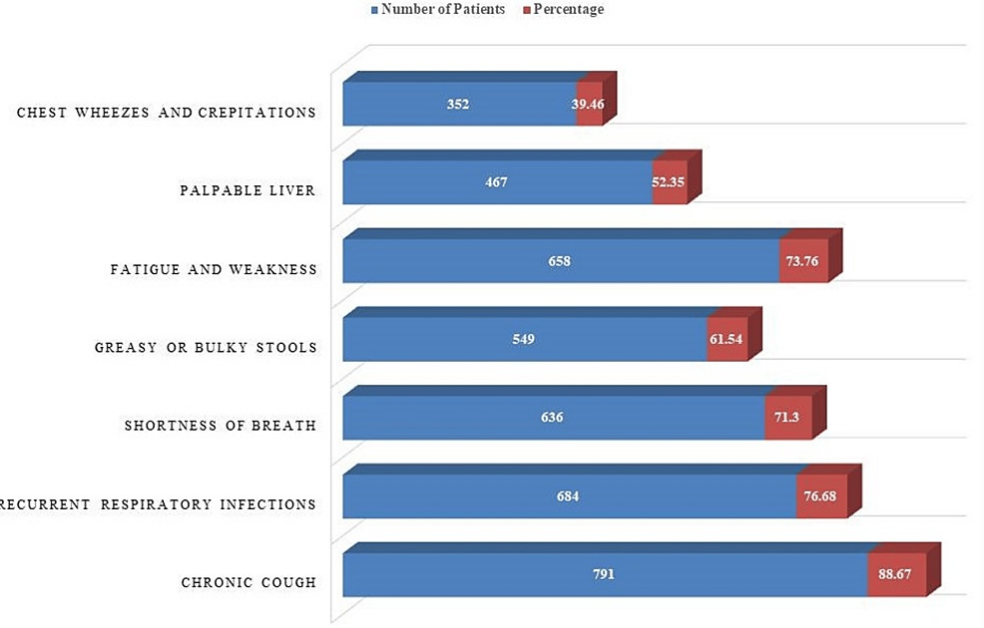
Prevalence of clinical manifestations and symptoms in cystic fibrosis patients in Pakistan

According to the clinical manifestations and symptoms, chronic cough was the most prevalent symptom, affecting 88.67% of the patients (n=791) (Figure 1). Recurrent respiratory infections were also common, occurring in 76.68% of the cases (n=684). Fatigue and weakness were reported by 73.76% of the patients (n=658). Other significant symptoms included shortness of breath (n=636; 71.3%), greasy or bulky stools (n=549; 61.54%), palpable liver (n=467; 52.35%), and chest wheezes and crepitations (n=352; 39.46%).

According to the frequency of complications observed among CF patients in Pakistan, pulmonary infections were the most prevalent complication, affecting 22% of the patients (n=197), followed by CF-related diabetes (n=187; 21%) and malabsorption (n=178; 20%). Airway obstruction/inflammation was seen in 14% of patients (n=125) while gastrointestinal complications were found in 10% of cases (n=89). Liver disease was observed in 9% of patients (n=80), and hepatobiliary involvement had the lowest frequency at 4% (n=36) (Table 2).

**Table 2:** Complication profile of cystic fibrosis patients in Pakistan

Complication	Number of patients (n)		Percentage (%)
Pulmonary infections	197		22
CF-related diabetes	187		21
Malabsorption	178		20
Airway obstruction/inflammation	125		14
Gastrointestinal complications	89		10
Liver disease	80		9
Hepatobiliary involvement	36		4

A comprehensive analysis was conducted on a set of 892 patient samples to detect mutations in cystic genes. From this study, a total of 12 mutations were successfully identified, namely, R117H, F508del, G85E, 2183AA>G, 2789+5G>A, W1282X, 3272-26A, N1303K, A455E, 2184delA, R334W, and 3849. The most common mutation is F508del, with 41 patients (14.91%) having a heterozygous gene mutation and 28 patients (10.19%) having a homozygous gene mutation, making up a total of 69 patients. Another notable mutation is N1303K, with 34 patients (12.38%) having a heterozygous gene mutation and 16 patients (5.82%) having a homozygous gene mutation, totaling 50 patients (Table 3). The associated asymptotic significance values for both groups are 1.000 and 0.998, respectively (Table 3). These results suggest that there is no significant difference between the two groups, as the p-values are close to 1, indicating that any observed differences are not statistically meaningful.

**Table 3:** Frequency analysis of cystic fibrosis gene mutations in a patient population n = Frequency of cystic fibrosis gene mutations in patients P-value <0.05 was considered significant.

Mutation	Heterozygous gene mutant			Homozygous gene mutant			Total	
	Number (n)	Percentage (%)	P value	Number (n)	Percentage (%)	P-value	n	%
F508del	41	14.91	1.000	28	10.19	0.998	69	25.1
N1303K	34	12.38		16	5.82		50	18.2
G85E	27	9.83		19	6.91		46	16.74
W1282X	19	6.9		9	3.28		28	10.18
A455E	15	5.47		3	1.09		18	6.56
2183AA>G	13	4.72		2	0.72		15	5.44
2184delA	8	2.9		0	0		8	2.9
3849	10	3.64		0	0		10	3.64
2789+5G>A	0	0		13	4.72		13	4.72
R334W	0	0		7	2.54		7	2.54
3272-26A	1	0.36		4	1.45		5	1.81
R117H	4	1.45		2	0.72		6	2.17
Total	172	62.56		103	37.44		275	100

**Figure 2:**
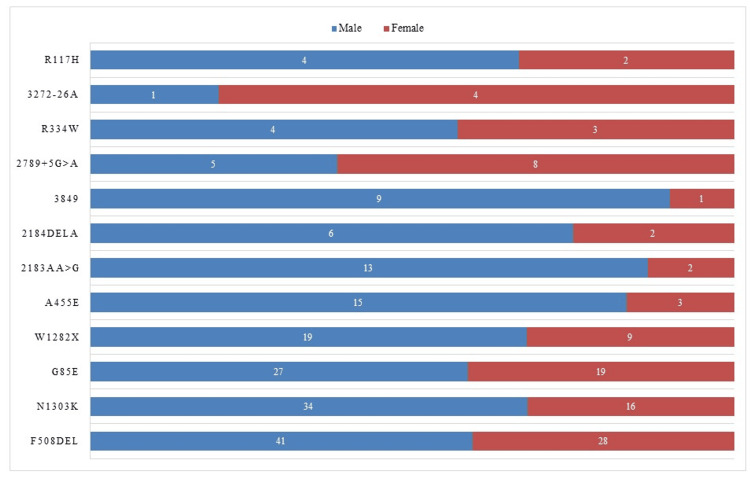
Distribution of CFTR gene mutations by gender in cystic fibrosis patients in Pakistan

The distribution of CFTR gene mutations among male and female cystic fibrosis patients in Pakistan reveals interesting patterns. The most common mutation observed is F508del, with 41 (4.59%) cases in males and 28 cases (3.13%) in females, totaling 69 cases (7.73%), which was followed by the N1303K mutation, with 34 cases (3.81%) in males and 16 cases (1.79%) in females, making a total of 50 cases (5.60%) (Figure 2).

Table 4 presents the results of a paired samples t-test conducted to compare the mean differences between two related groups: "Heterozygous gene mutant" and "Homozygous gene mutant." The mean difference in the paired observations is 5.750, with a standard deviation of 9.147 and a standard error of the mean at 2.640. The 95% confidence interval of the difference ranges from -0.061 to 11.561. The t-value is 2.178 with 11 degrees of freedom, and the associated two-tailed p-value is 0.052. While the p-value is slightly above the conventional significance level of 0.05, there is a trend toward a significant difference, suggesting that with a larger sample size, this difference may become statistically significant.

**Table 4:** Paired samples t-test results comparing mean differences between heterozygous gene mutant and homozygous gene mutant groups df: degrees of freedom, Std. Deviation: standard deviation, CI: confidence interval of the difference, Sig. (2-tailed): This represents the significance level associated with a two-tailed statistical test. It indicates the probability of obtaining the observed results (or more extreme results) if there were no true differences between the groups being compared.

Variable	Paired Differences					t	df	Sig. (2-tailed)
	Mean	Std. Deviation	Std. Error Mean	95% CI				
				Lower	Upper			
Heterozygous gene mutant - Homozygous gene mutant	5.750	9.147	2.640	-.061	11.561	2.178	11	.052

## Discussion

The study aimed to provide a comprehensive understanding of the demographic and genetic characteristics of the patients, the prevalence of various clinical manifestations and symptoms, the frequency of complications, and the distribution of CFTR gene mutations within the studied population of Pakistan.

The study's significant discovery of a high frequency of consanguinity (n=691; 77.47%) among CF patients in the Pakistani population highlighted a fascinating feature of CF. Comparisons with earlier research show that Pakistan has higher consanguinity rates than certain other countries [10,11]. This finding suggests that the increased incidence of CF in Pakistan could be influenced by a combination of genetic and hereditary factors, along with cultural practices related to consanguineous marriages.

The clinical manifestations and symptoms reported by CF patients in this study were consistent with previous findings [12,13]. Chronic cough, recurrent respiratory infections, and fatigue were the most prevalent symptoms. These findings reinforce the well-known respiratory complications associated with CF and highlight the need for targeted interventions to manage these symptoms effectively.

The study's findings contribute to the body of knowledge surrounding CF symptoms and provide valuable insights for improving patient care and treatment strategies. Regarding complications, pulmonary infections were the most common, followed by cystic fibrosis-related diabetes and malabsorption. These findings are consistent with previous studies [14-16], indicating that CF patients globally experience similar complications. It underscores the importance of comprehensive care and monitoring to address these complications promptly and prevent further health deterioration.

The analysis of CFTR gene mutations revealed several notable mutations, with F508del being the most frequent mutation observed in this study. These findings were consistent with global data [17,18], as F508del is the most common CFTR mutation worldwide. The presence of other mutations, such as N1303K and G85E, also aligns with previous study [19]. These similarities in mutation frequencies suggest a shared genetic profile of CF in the Pakistani population. Comparing the distribution of CFTR gene mutations between males and females showed similar patterns, with F508del and N1303K being the most prevalent mutations in both genders. These findings were consistent with previous studies [19,20], indicating that certain CFTR mutations are gender-independent and contribute to CF development in both males and females.

Limitations

The limitations of this research include limited sample size, a focus on common mutations without exploring novel ones, a lack of in-depth clinical data, and potential statistical constraints due to sample size. These limitations should be considered in interpreting the findings and planning future investigations.

## Conclusions

This study provided valuable insights into the frequency of CF gene mutations, clinical manifestations, and complications within the population of Pakistan. The findings highlight the high prevalence of consanguinity among CF patients in Pakistan, suggesting a possible genetic influence on the disease. The study also confirms the common clinical symptoms and complications associated with CF, emphasizing the need for targeted interventions and comprehensive care. Furthermore, the analysis of CFTR gene mutations reveals the presence of commonly observed mutations worldwide, such as F508del and N1303K, suggesting a shared genetic profile in the Pakistani population. These findings contribute to the existing knowledge of CF in Pakistan and provide valuable insights for healthcare professionals, enabling them to improve diagnosis, management, and genetic counseling for CF patients.
